# Learning without Borders: A Review of the Implementation of Medical Error Reporting in Médecins Sans Frontières

**DOI:** 10.1371/journal.pone.0137158

**Published:** 2015-09-18

**Authors:** Leslie Shanks, Karla Bil, Jena Fernhout

**Affiliations:** Médecins Sans Frontières-Operational Centre Amsterdam, Plantage Middenlaan 14, PO Box 10014, 1001 EA Amsterdam, The Netherlands; University of California San Diego, UNITED STATES

## Abstract

**Objective:**

To analyse the results from the first 3 years of implementation of a medical error reporting system in Médecins Sans Frontières-Operational Centre Amsterdam (MSF) programs.

**Methodology:**

A medical error reporting policy was developed with input from frontline workers and introduced to the organisation in June 2010. The definition of medical error used was “*the failure of a planned action to be completed as intended or the use of a wrong plan to achieve an aim*.” All confirmed error reports were entered into a database without the use of personal identifiers.

**Results:**

179 errors were reported from 38 projects in 18 countries over the period of June 2010 to May 2013. The rate of reporting was 31, 42, and 106 incidents/year for reporting year 1, 2 and 3 respectively. The majority of errors were categorized as dispensing errors (62 cases or 34.6%), errors or delays in diagnosis (24 cases or 13.4%) and inappropriate treatment (19 cases or 10.6%). The impact of the error was categorized as no harm (58, 32.4%), harm (70, 39.1%), death (42, 23.5%) and unknown in 9 (5.0%) reports. Disclosure to the patient took place in 34 cases (19.0%), did not take place in 46 (25.7%), was not applicable for 5 (2.8%) cases and not reported for 94 (52.5%). Remedial actions introduced at headquarters level included guideline revisions and changes to medical supply procedures. At field level improvements included increased training and supervision, adjustments in staffing levels, and adaptations to the organization of the pharmacy.

**Conclusion:**

It was feasible to implement a voluntary reporting system for medical errors despite the complex contexts in which MSF intervenes. The reporting policy led to system changes that improved patient safety and accountability to patients. Challenges remain in achieving widespread acceptance of the policy as evidenced by the low reporting and disclosure rates.

## Background

Medical error reporting is an essential part of a patient safety framework within any health care organization [1,2,3]. Medical error reporting systems help identify risks and vulnerabilities in systems in order to implement improvements. Simply put, they help reinforce systems that ‘make it easier to do the right thing and harder to do the wrong thing’.

There are considerable challenges working in resource-limited settings particularly during humanitarian interventions. Many of these factors increase vulnerability to errors that impact on quality of care. These factors include the emergency nature of the response, high patient loads, a high level of patient severity and degree of acuteness, health worker shortages leading to a reliance on lay staff or poorly trained staff, lack of sophisticated monitoring and diagnostic devices, complexity of care, insecurity, and language and cultural barriers. Médecins Sans Frontières has invested considerably in the standardization of approaches to emergencies, including customized protocols and procedures, training, quality control, and monitoring systems to track patient outcomes [4,5]. All are designed to improve the quality and speed of the intervention and to reduce the risk of mistakes. Nevertheless, errors still occur.

MSF-Operational Centre Amsterdam (MSF) decided to implement a medical error reporting system prompted by two motivations. One was as part of a strategy to bring the science of patient safety into the organization linked to on-going efforts to improve the quality of care delivered. The second was to improve MSF’s accountability to patients. This latter objective was prompted by two patient complaints, through which it became clear that some MSF staff were not adequately prepared to deal with patients when things go wrong. One case involved the late recognition of an adverse effect of a medication. The other involved delayed management of obstructed labour. In both cases, there were obvious deficits in communication once the errors had been discovered such that the patients felt they needed to escalate the issue as they had either lost faith in the medical team and/or had failed to receive adequate compensation.

This paper reports on three years of medical error reports in the organization to analyse the type and impact of errors reported, and describes where the reported errors occurred. It also aims to look at the broader question of feasibility of instituting such a policy. To our knowledge it is the first report of a voluntary incident reporting system in both resource-limited settings and during humanitarian interventions.

## Methods

### Ethical Review

Data were collected without personal identifiers and entered anonymously into a database. The study met the standards set by the MSF Ethics Review Board for retrospective analysis of routinely collected programmatic data [6].

### Policy Development

A medical error reporting policy was developed in 2010 using standard references such as the Institute of Medicine (IOM) Report: *To Err is Human* and WHO resources on error reporting. [1,2] The definition of medical error used was that of the IOM, namely “*the failure of a planned action to be completed as intended or the use of a wrong plan to achieve an aim*.” A consultation was held with senior field staff to get their feedback prior to finalizing the policy. The policy was introduced in an overall framework of patient safety, with clear organizational learning objectives. Field teams were asked to report all medical errors and near-miss events to headquarters using a standardized format (see S1 Text). Individual names of medical staff involved with the error were not required though their professional cadre was requested (e.g. pharmacist, nurse). Identification of the project site was requested. The policy promoted active disclosure of the medical errors to patients and families as well as to health authorities. Reporters were asked to describe the root cause of the error, and list the remedial actions put in place to prevent a similar error in the future. No additional resources, either financial or human resources, were added to implement the policy.

### Dissemination and promotion of the policy

The policy was introduced at the annual meeting of medical coordinators and followed up by regular email communication. A package of tools from the WHO Patient Safety website as well as specific resources on how to disclose errors was disseminated electronically and on DVD to field staff along with the policy. Details of the composition of the resource package can be found in S2 Text.

Patient safety and medical error reporting were introduced as standard modules on MSF management training courses. Examples of the errors reported with key learning points highlighted were sent regularly to field staff. Annual meetings of senior field staff (medical coordinators and managers) included sessions on medical error that were supported by the presence of a medical ethicist. Internal newsletters highlighted system issues identified through the reporting system. Efforts were made to include the policy in briefings for new staff.

### Root Cause Analysis

Root cause analysis (RCA), a technique whereby the investigation and analysis of the incident is used to identify a root cause or causes of the error, was taught to headquarters staff and field-based medical coordinators. Headquarters staff was encouraged to model RCA investigations while on field trips to improve the skills of field staff.

### Collection of reports

Reports were sent to headquarters by email. The headquarters-based Health Advisor (either MD or RN) reviewed all reports for his/her countries with the support of the headquarters’ specialist advisors (e.g. surgeon, paediatrician, pharmacist). Where information was missing, the Health Advisors were encouraged to follow up with the field teams by email or phone. Final arbitration on whether or not an error met the definition of a medical error was done by the Medical Director (LS). Feedback was sent to field teams on the report and the remedial actions proposed.

### Analysis

Error reports were entered anonymously into an Excel database. Errors were classified using the typology from the IOM report derived from Leape et al. [7]. The typology was adapted with the addition of a medical supply category. This category included any breakdown in the supply chain that touched (or potentially could touch) the patient. It included events such as use of expired test kits and breaks in the cold chain. Where more than one category was possible, the more serious error was chosen based on the hierarchy described by Leape et al. [7]. The hierarchy prioritised 4 categories (errors or delays in diagnosis, technical errors, drug administration errors and inappropriate treatment) over the other categories. The impact of the error on the patient was coded using the National Coordinating Council (NCC) for Medication Error Reporting and Prevention classification system [8]. Classification of incidents was done by one of the authors (LS) and reviewed by another (JF). Discordant judgements were resolved by consensus discussion.

### Setting

The policy was disseminated to all field projects of MSF. Over the study period, MSF was present in 19–23 countries with an average of 60 projects operating at any one time. New projects and country missions were opened and others closed over the reporting period, such that each reporting year saw a project turnover of approximately 20%. The countries and projects therefore represent a mix of long-term and newer projects. Each project was composed of one or more of the following program sites: hospitals, outpatient clinics, community outreach, therapeutic feeding centres, outbreak response (e.g. cholera treatment centres) and/or specialised disease programs (e.g. HIV, TB, visceral leishmaniasis). Examples of the larger emergency responses that took place in the study period are the Haiti Earthquake and subsequent cholera outbreak, nutritional crisis in the Horn of Africa, conflict in South Sudan and the Democratic Republic of Congo, and floods in Pakistan. The most common model of intervention was MSF support to existing Ministry of Health (MoH) facilities. In a minority of settings, MSF was solely responsible for the management and functioning of the health facility. All care was provided free of charge.

## Results

A total of 179 errors were reported between June 2010 and May 2013 from 38 projects in 18 countries. Countries reporting were in Africa (South Sudan, Nigeria, Ivory Coast, Democratic Republic of Congo, Central African Republic, Chad, Somalia, Ethiopia, Zimbabwe, Swaziland), Asia (India, Bangladesh, Pakistan, Afghanistan, Myanmar), Latin America (Colombia, Haiti) and Oceania (Papua New Guinea). Countries without any reports were Iraq (mental health programme), Tajikistan (paediatric multi-drug resistant tuberculosis), Russia (mental health and tuberculosis), and Yemen (emergency room and primary care).

Projects reported a median of 2 [range 1–66] errors. Sixteen projects reported only one error. The projects with the most reports were found in South Sudan and Pakistan.

The rate of reporting was 31, 42, and 106 incidents/year for programme years 1, 2 and 3 respectively.

Seven reports (3.9%) came from the first six months of an emergency intervention. Adults represented 85 of 134 (63.4%) patients affected. Amongst the 49 children, 14 (28.6%) were less than one year old, and 29 (59.1%) less than five years old. The male: female ratio was 0.42.

Overall, the most common sites of the errors reported were the medical wards of the hospital in 53(29.6%) cases, maternity ward in 34(19.0%) cases and outpatients department (OPD) in 18 (10.1%). Looking at the subset of 105 errors that occurred in hospital, 50.5% occurred in the medical wards, 32.4% in the maternity ward, 13.3% in surgery, and 3.8% in the Emergency Room. Further characteristics of the errors can be found in Table 1.

**Table 1:** Characteristics of Errors Reported.

Time to recognise error		N (%)
	<24 hours	47 (26.3)
	> = 24 hours but still under care	64 (35.8)
	After discharge from care/death*	50 (27.9)
	Data missing	18 (10.1)
Individual identifying error	Member of immediate health team	104 (58.1)
	Supervisor	47 (26.3)
	Headquarters staff	5 (2.8)
	Family member/patient	5 (2.8)
	Data missing	18 (10.1)
Location of error	Medical ward	53 (29.6)
	Maternity	34 (19.0)
	Outpatients department	18 (10.1)
	Therapeutic feeding centre	15 (8.4)
	Surgery	14 (7.8)
	Emergency Room	4 (2.2)
	Community	3 (1.7)
	Other	36 (20.1)
	Data missing	2 (1.1)
Cadre of staff judged most responsible	Multiple cadres**	66 (36.9)
	Physicians	34 (19.0)
	Lay workers	30 (16.8)
	Nurses	22 (12.3)
	Midwives	11 (6.1)
	Lab technicians	4 (2.2)
	Pharmacists	3 (1.7)
	Logistician	2 (1.1)
	Other	2 (1.1)
	Data missing	5 (2.8)

*An example of an error discovered after discharge from care is that of a retained surgical sponge causing a post operative infection that presents after discharge from hospital or a medication error that is recognised after an outpatient has been discharged from the TB programme.

** An example of an error involving multiple cadres of staff is a failure to monitor a patient, resulted in delayed recognition of sepsis where the responsibility is with both the nursing staff and the physicians.

The majority of errors were categorized as dispensing errors (62, 34.6%), errors or delays in diagnosis (24,13.4%) and inappropriate treatment (19, 10.6%) (Table 2). Amongst the 106 errors that took place in hospital, the most common types of errors were dispensing errors (26, 24.5%), errors or delays in diagnosis (18, 17.0%) and inappropriate care (16, 15.1%) as described in Table 3.

**Table 2:** Types of Errors Reported.

Category	Description	Example	Total
Diagnosis	Error or delay in diagnosis	Delayed recognition of septic arthritis	24 (13.4)
	Failure to employ indicated tests	Switch to second line anti-retrovirals in an HIV patient without repeating the second viral load test to confirm treatment failure	2 (1.1)
	Use of outmoded tests or therapy		0
	Failure to act on results of monitoring or testing	Failure to adjust tuberculosis treatment when sputa returned positive at month 4 of first line treatment.	7 (3.9)
Treatment	Avoidable delay in treatment or in responding to an abnormal test	Delay in recognition and response to fetal distress during labour.	15 (8.4)
	Technical error in the performance of an operation, procedure or test	Prostatic urethral injury following catheterisation.	14 (7.8)
	Inappropriate (not indicated) care	Woman in labour received medication to augment labour without a clear indication and in the presence of contraindications.	19 (10.6)
	Error in the administering of the treatment	Error made in crossing and typing a blood transfusion resulting in a transfusion reaction.	12 (6.7)
	Error in the dose or method of using a drug (dispensing error)	Scheduled dose of intravenous antibiotics was missed.	62 (34.6)
Medical Supply	Error in the procurement or storage of a test device or medication	Supply rupture of hepatitis B tests resulting in inability to screen blood transfusions.	5 (2.8)
Preventive	Inadequate monitoring or follow up of treatment	No vital signs recorded on chart for child in the in-patient feeding programme.	13 (7.3)
	Failure to provide indicated prophylactic treatment	Failure to regularly turn a semi-comatose patient resulting in pressure ulcers	1 (0.6)
Other	Failure in communication	Lack of clarity in communicating division of responsibilities during hand-over.	4 (2.2)
	Equipment failure	Autoclave used to sterilise surgical instruments was identified with a malfunction that may have impacted on sterility.	1 (0.6)
	Other system failures		0
**TOTAL**			**179 (100%)**

**Table 3:** Type of Error Classified by Site where Error Occurred.

	Description	Hospital N (%)	OPD*N (%)	TFC**N (%)	Community N (%)	Other N (%)	Total
Diagnosis	Error or delay in diagnosis	18 (17.0)	1 (5.6)	3 (21.4)	0	2 (5.6)	**24**
	Failure to employ indicated tests	0	2 (11.1)	0	0	0	**2**
	Use of outmoded tests or therapy	0	0	0	0	0	**0**
	Failure to act on results of monitoring or testing	2 (1.9)	2 (11.1)	0	0	3 (8.3)	**7**
Treatment	Avoidable delay in treatment or in responding to an abnormal test	10 (9.4)	4 (22.2)	0	0	1 (2.8)	**15**
	Technical error in the performance of an operation, procedure or test	13 (12.3)	0	0	0	1 (2.8)	**14**
	Inappropriate (not indicated) care	16 (15.1)	0	1 (7.1)	0	2 (5.6)	**19**
	Error in the administering of the treatment	7 (6.6)	2 (11.1)	1 (7.1)	0	2 (5.6)	**12**
	Error in the dose or method of using a drug (dispensing error)	26 (24.5)	5 (27.8)	5 (35.7)	1 (33.3)	24 (66.7)	**61**
Medical Supply	Error in the procurement or storage of a test device or medication	1 (0.9)	2 (11.1)	0	2 (66.7)	0	**5**
Preventive	Inadequate monitoring or follow up of treatment	8 (7.5)	0	4 (28.6)	0	0	**12**
	Failure to provide indicated prophylactic treatment	1 (0.9)	0	0	0	0	**1**
Other	Failure in communication	3 (2.8)	0	0	0	1 (2.8)	**4**
	Equipment failure	1 (0.9)	0	0	0	0	**1**
	Other system failures	0	0	0	0	0	**0**
**TOTAL**		**106**	**18**	**14**	**3**	**36**	**177**

*Outpatient Department

**Therapeutic Feeding Centre

Total number of reports is 177 due to two cases with missing data.

The impact of the error was categorized as no harm (58, 32.4%), harm (70, 39.1%), death (42, 23.5%) and unknown (9, 5.0%) as detailed in Table 4.

**Table 4:** Classification of the Impact of Errors. Coding of errors is based on the National Coordinating Council for Medication Error Reporting and Prevention classification system.

Classification	Category	Description	N (%)
No error	A	Circumstances or events that have the capacity to cause error	NA
No harm	B	An error occurred but the error did not reach the patient	2(1.1)
	C	An error occurred that reached the patient but did not cause the patient harm	33(18.4)
	D	An error occurred that reached the patient and required monitoring to confirm that it resulted in no harm to the patient and/or required intervention to preclude harm	23(12.8)
Harm	E	An error occurred that may have contributed to or resulted in temporary harm to the patient and required intervention	35(19.6)
	F	An error occurred that may have contributed to or resulted in temporary harm to the patient and required initial or prolonged hospitalization	16(8.9)
	G	An error occurred that may have contributed to or resulted in permanent patient harm	17(9.5)
	H	An error occurred that required intervention necessary to sustain life	2(1.1)
Death	I	An error occurred that may have contributed to or resulted in the patient’s death	42(23.5)
UNKNOWN			9(5.0)
TOTAL			179

Disclosure to the patient or the family occurred in 34 cases (19.0%), did not take place in 46 (25.7%), and was not applicable in 5 (2.8%) cases. Data was missing for the remainder (94, 52.5%). Not applicable for disclosure refers to errors where the individuals affected could not be identified or where the family or patient had themselves notified the health staff of the error. Classifying disclosure status by impact of the error shows a trend toward a higher percentage of disclosure as the severity of the impact increased as can be seen in Table 5.

**Table 5:** Disclosure Status Classified by Impact of Error. Errors where the impact is not known are excluded, as are errors where disclosure was classified as not applicable.

	Disclosure N (%)			
Impact	Yes	No	Missing Data	Total
No Harm	3 (5.2)	9 (15.5)	46 (79.3)	58
Harm	19 (27.5)	11 (15.9)	39 (56.5)	69
Death	12 (30.0)	25 (62.5)	3 (7.5)	40
Total	34 (20.4)	45 (27.0)	88 (52.7)	167

At least ten of the errors prompted a change in practice at headquarters. These included changes in medical protocols and guidelines and to medical supply procedures. The remainder of the remedial actions was at field level and included improved training, enhanced supervision, and changes in staffing, nursing practice, and laboratory procedures. As medication error was the most common category of error reported, a number of remedial actions were linked to pharmacy management. These included wall charts and other memory aids to ensure dosages were correct, especially where nursing staff had little pharmacology training and were not used to double-checking dosages or challenging prescriptions written by more senior staff. In some cases, the pharmacy and ward stock required re-organization to ensure look or sound-alike drugs were stored separately and clearly labeled. This was particularly important for sites where trained lay workers dispensed or administered medications in the absence of qualified staff. Examples of errors and remedial actions chosen to be illustrative of the errors reported can be found in Table 6.

**Table 6:** Examples of Errors Reported.

1.	In Country X, lay workers administer vaccinations at a hospital in a remote region of the country. On this occasion, the physician was urgently consulted about a patient with a presumed anaphylactic reaction to a tetanus toxoid injection. The patient was successfully resuscitated, but when symptoms recurred after several hours, the physician looked further. It was discovered that a lay worker had mistaken a vial of insulin for tetanus toxoid when loading the cold box from the refrigerator. In total, 6 patients had received the insulin injections over 2 days. All were traced, informed, and monitored for blood glucose levels. All recovered without evidence of permanent injury.
	The team realised that the mistake was made because insulin and vaccines were both stored on the same shelf of the refrigerator. They revised procedures so that a registered nurse would dispense the vaccine vials. In addition, they changed the packing of the refrigerator so that insulin and other injectable medications were stored separately from the vaccines.
2.	The nurse on duty admitted a woman to hospital complaining of back pain and weakness. The admission physical made note of a tender abdomen. On the 4^th^ day of admission, she died on the ward.
	The case was flagged on Mortality Review, as the chart was remarkable for the fact that there was no documentation from a physician. It was therefore difficult to assess the cause of death, however an unrecognised acute abdomen was suspected. The remedial plan included new signage on each ward to identify the daily responsible physician, reinforcing the importance of a physician review for all admissions along with good documentation on the patient record. Finally the incident led to the decision to open a position for an additional expatriate physician to address workload issues at the level of supervision of the medical team.
3.	A woman in her third trimester of pregnancy was admitted to hospital with anaemia (haemoglobin 4.5 g/dL) for transfusion. She received the first unit without incident, but immediately upon the hanging of the second unit she complained of chills and chest pain. The nurse immediately stopped the infusion, and called the physician. The physician administered the appropriate medications for a transfusion reaction and the symptoms abated.
	The laboratory supervisor investigated and found that the donour blood bag was labelled incorrectly for the blood group. In addition to reinforcing procedures in the laboratory, the nursing staff was reminded to do bedside blood grouping prior to all transfusions. An order for the bedside grouping cards was made and training provided to the staff.

## Discussion

An error reporting system proved feasible to implement without requiring additional human or financial resources. The number of reports was low but increased over time through a variety of strategies to improve implementation of the policy (Table 7). It resulted in changes to improve practice at both field and headquarters level. Most reported events resulted in either temporary or permanent harm. The high proportion of events leading to harm may reflect reporting bias, as serious incidents were more likely to come to the attention of staff and supervisors. Recognition and reporting of the error was most often done by members of the immediate health team, indicating a level of support for reporting from frontline workers.

**Table 7:** Strategies to Improve Reporting.

1	Incident reporting systems should be implemented as part of a broad organization-wide focus on patient safety.
2	Champions in the form of senior medical staff at both field and headquarters are key to promoting the organizational change that incident reporting requires.
3	The inclusion of the policy in the induction package for new staff helps disseminate the policy and allows for discussion during briefings. It also avoids ‘surprising’ staff with the policy after an incident has occurred, when stress levels are high.
4	Management teams must be engaged early in order to assess the operational implications of the policy. A risk assessment on potential risks of reporting and disclosure of errors along with mitigation strategies should be prepared in advance, which can be adapted to the specifics of incidents as they occur.
5	Encouraging reporting of near misses and errors that did not cause harm and/or introducing ‘reporting weeks’ can help teams become familiar with reporting
6	Root cause analysis requires training and ongoing support from experts, but when done well can improve the learning experience for staff, thereby reinforcing the value of reporting.
7	Labeling the reporting system as ‘avoidable medical incidents’ avoids potentially emotive terms such as ‘medical error’.
8	Field staff benefit from the sharing of positive examples of reporting from their peers in other sites, in addition to the learning that comes with the sharing of remedial actions.
9	Reporting on the implementation of the policy as part of routine organizational progress reporting helps embed incident reporting in standard organizational procedures, and promotes visibility of the policy for both frontline staff and supervisors.
10	Engagement of partner Ministries of Health is important to ensure a shared understanding of the objectives of the reporting system.
11	Psychological support for staff involved in serious errors, in addition to that provided for the patient and families affected, should be routinely offered and encouraged where necessary. Staff health programs are important resources for this purpose.
12	Demonstrating organizational change in response to incident reporting is critical to maintaining the trust of staff to continue reporting.

The commonest types of errors reported were medication errors. These involved prescription errors as well as mistakes made dispensing or administering medication. Most errors took place in hospitals, specifically medical wards, followed by maternity wards. Looking only at cases that occurred in hospitals, a relatively small proportion involved surgical care, in contrast to other reports of hospital-based surveillance of events in the literature [9,10,11]. This may be due to relatively low surgical volumes as the surgical programs tended to focus on emergency and urgent cases rather than electives, or other unknown factors. The reporting of surgical incidents served as an opportunity to reinforce use of the WHO Safe Surgery Checklist [12]. Less than one in 20 errors was reported from projects in the first six months of an emergency intervention. While emergency settings are highly vulnerable to medical error due to their complexity, extreme limitation of resources and changeover of staff, they are also less likely to produce voluntary reports due to high workload and the prioritization of direct aid delivery.

### Reporting Rates

A challenge for voluntary reporting systems is to increase the reporting rate. Our rates did improve over time, but remained low. Some projects and countries did not report at all during the course of the period. These tended to be smaller projects with lower volumes; nevertheless, the lack of policy uptake is concerning. Other projects reported often, in particular several projects that adopted a strategy to report low impact errors on a regular basis in order to familiarize the team with reporting. This is similar to “reporting weeks” that have been reported to improve voluntary reporting rates [13]. Champions of the reporting system proved to be critical, and likely contributed to much of the variation amongst projects.

It is not possible to calculate to what degree under-reporting was taking place as incident reporting systems do not allow estimates of rates due to lack of robust definitions for incidents, lack of reliable data for the population at risk and the absence of a surveillance system [3]. There are no published reports from resource limited settings or humanitarian interventions that report data collected from voluntary incident reporting systems that can serve as comparison. Further, error-reporting systems are known to represent only a fraction of the errors actually occurring, with at most, 10% of errors captured in such systems [14].

The standard for determining rates of errors or adverse events is retrospective chart review. Kalra and colleagues summarized the major reports of hospital based adverse events that used this methodology and found incident rates to vary from 3% to 36% [15]. Fatality rates ranged from 2% to 21% amongst the errors identified. Figures for the developing world are more difficult to determine though two large WHO sponsored studies of hospitals do exist for developing and transitional economies. One study analysed records from 26 hospitals in eight countries in the Eastern Mediterranean and Africa regions [11]. The authors identified 8.2% records with at least one adverse event, and judged 83% of these events to be preventable. Amongst these, 30% were judged to contribute to death. The second study took place in 58 hospitals in 5 countries in Latin America [16]. The incidence of adverse events was 20% with close to 60% judged to be preventable. Death resulted in 6%. The samples in both studies included mainly large teaching and urban hospitals and therefore are not likely representative of hospitals in low resourced settings or humanitarian settings.

A Cochrane review looked at interventions to improve reporting of adverse clinical events. However the overall quality of evidence was judged so low that they were unable to draw any conclusions [17]. In discussions with field staff, we were able to identify a number of barriers to reporting. One was a reluctance to admit mistakes. This attitude was found across all countries, but was particularly a challenge in countries with authoritarian states and in cultures where a high value was attached to ‘saving face’. A partial solution was to refer to preventable medical incidents to avoid the pejorative connotation of an error. Education was used to reinforce the fact that most errors are a result of system failures rather than individual mis-steps.

Many staff stated they felt a sense of responsibility for the work MSF was doing, often as the only or main health provider for populations with high needs. This responsibility was sometimes felt to be in conflict with the duty to report, and they worried that being open about an error would have a negative impact on MSF’s acceptance within the community or with authorities. They also experienced conflict between their good intentions as both humanitarians and health care workers, and the reality that a patient or patients had been harmed by an error. This highlights the importance of providing psychological support for health care workers involved in a medical error incident.

As has been reported elsewhere, we found that de-linking reporting with disciplinary action was important to acceptance of the reporting system [18,19]. On several occasions reporting was used in a punitive fashion, and it was necessary to address this quickly. In the specific case of suspected negligence by a staff member, it was difficult in practice to separate the reporting from human resources management and therefore there were rare occasions where disciplinary action resulted. Our classification system did not distinguish between errors due to neglect and those due to other factors, so we are not able to quantify how often negligence occurred. However, in cases where negligence was felt to have occurred, teams were still encouraged to do the root cause analysis in order to determine if factors such as supervision, training, or workload could reduce the risk or impact of negligent actions. Finally, rare cases elicited questions of professional competency to work in the complex environments where MSF operates, and prompted development of a formal procedure for dealing with allegations of incompetency separate to the reporting system.

Another barrier to reporting was the challenge of ensuring that staff was aware of the policy given the high turnover of staff working in dozens of countries and in hundreds of different facilities. We tried to address this by including the error policy as a standard part of the briefing of new staff. Lack of time to report was also a factor as individuals were often overloaded dealing with urgent cases and chronically high workloads. Reporting of near miss events was promoted [20] but as evidenced by the low number reported (two cases) this was not successful.

### Disclosure and compensation

Disclosure to patients and families was challenging, with only 40% of incidents with information recorded stating that disclosure took place. There was a large proportion of missing data, and it is likely that in most of these cases disclosure did not take place. However, these figures hide a number of incidents where disclosure did take place successfully in extraordinarily difficult contexts. Despite a large body of work in the West on patient perceptions of disclosure and how to disclose, there is a knowledge gap for non-Western contexts particularly those from low resourced settings. Nevertheless, the work that has been done serves to confirm that patients want to be told about medical errors [21–23].

Many of the reasons not to disclose were common to those found in better resourced and more stable settings [24,25] and had considerable overlap with the reporting barriers already discussed. In addition, fear of litigation or legal consequences was common. Fortunately, in the period under review MSF did not experience any legal action linked to the policy of disclosure. However many countries where MSF works do not have legislation protecting health care workers from reporting, and this is an important point of attention for the future.

There were unique considerations making disclosure, whether to patients or to health authorities, particularly challenging. These related to the specific contexts of war and instability where MSF works and include physical security of teams and reprisals from authorities or communities that could result in MSF losing the space to work in an area. The policy stated that in cases where either physical safety of staff or the operational viability of the project is threatened, it might be appropriate not to disclose. The decision must be referred to headquarters and requires a detailed risk assessment. To date, there have been no cases where on review it was felt justified not to disclose. These discussions however were difficult, and we learned to encourage the project managers to do a generic risk assessment for their setting prior to implementing the policy. This allowed a more reflective, less emotionally charged analysis of the risks to deal with disclosure of an incident than an analysis done immediately after a serious event had occurred. It also provided an opportunity to develop mitigation strategies that could be immediately activated following the occurrence of an incident.

Compensation in the form of money or payment of expenses, to the individual or family harmed by the medical error was not reported consistently, however anecdotally it appeared that only a small minority of cases received compensation. Some managers were concerned about ‘setting a precedent’ in providing compensation, and worried that all patients with adverse outcomes regardless of whether or not an error had occurred would expect compensation. We found that support from an external ethicist was very helpful in having these discussions with field teams.

### Involvement of the Ministry of Health

The World Health Assembly adopted a resolution on patient safety in 2002 that includes the need to report adverse events and near misses [26]. Subsequently the WHO has developed a number of resources to assist countries and organizations to implement reporting [2,27]. However our experience was that few of the local Ministries of Health we worked with were reporting errors, and many were not familiar with the principles. An example is when the use of expired vaccines was identified during a mass measles campaign. The error resulted in approximately 60 individuals receiving vaccine that was less than two months over the expiry date. The analysis of the error showed that the packing carton had vials with two different expiry dates, likely a result of re-packing after a previous vaccination campaign. MSF immediately notified the authorities. The health worker, employed by the Ministry of Health was fired, and the incident was used politically against MSF. The incident took months to be resolved, and clearly served as a disincentive to report and learn from future errors. This example points out the importance of discussing the policy with the local health authorities prior to implementation, in order to ensure they understand and support the objectives of the reporting system.

Despite this negative example, one of the unanticipated benefits of the policy was the opportunity to act as a catalyst for change through demonstrating a different way of dealing with medical errors with local health staff. The policy encouraged an open discussion of the incidents with the authorities to arrive at a jointly agreed strategy for remedial action. And as the errors could involve either MSF or MoH staff, it also helped foster an active learning culture between partners.

### Demonstrating Change

Critical to long-term acceptance of the reporting system, is to demonstrate change. We made efforts to ensure that the database of errors was disseminated regularly to all field missions. The remedial actions were emphasized, particularly when they involved change at headquarters, in order to highlight the shared responsibility for mistakes and to illustrate how reporting in an individual site can help all sites improve patient safety. Case studies were shared widely at trainings for all cadres of staff, again to ensure maximum dissemination of learning and to encourage reporting.

In the same spirit, it is hoped that this paper will encourage organizations working in resource limited settings or humanitarian contexts to institute incident reporting systems and to share the results. The experience detailed here is a start but much remains to be done. An online global database, as has been proposed in a recent commentary, could be one possible solution to increase the number and scope of events available for analysis [28]. However such a tool will succeed only with an organization-wide commitment to foster the culture change needed. Ultimately it is only through common recognition of the vulnerability of all health systems to error, along with an acceptance that good intentions are not enough, which will move this dialogue away from blame and towards improved safety for patients in low resourced and humanitarian settings.

### Limitations

The main limitation of interpretation of the data is the methodology, which does not allow calculation of error rates and is subject to reporting bias. In addition, our data is more challenging to interpret due to the wide variety of contexts, types of programming, and settings, which include a range from hospital-based care to community vaccination campaigns. It is therefore important to interpret our data as representative of the errors reported over the first 3 years of the programme, and not as representative of the errors that took place. Further we did not engage in a separate qualitative study to objectively assess the perceptions and experiences of individuals working with the reporting system. Finally common to reporting systems, it is difficult to measure the actual impact of the changes implemented on improved patient safety [3].

## Conclusion

It proved feasible to implement a voluntary reporting system of preventable medical incidents despite the challenging and diverse contexts where MSF works. The reporting protocol led to important changes that improved patient safety and accountability to patients. Significant challenges remain in achieving widespread acceptance of the policy as evidenced by the low reporting and disclosure rates. Priority areas for future research are to further understand the factors linked to unsafe medical care in low resourced and humanitarian settings in order to target quality improvement and to develop best practices on improving uptake of reporting in these complex environments.

## Supporting Information

S1 Text Reporting format. (DOCX)

S2 Text List of resources to support field implementation. (DOCX)
