# Supplementary material for: Learning without Borders: A Review of the Implementation of Medical Error Reporting in Médecins Sans Frontières
Source: PLoS One. 2015 Sep 18;10(9):e0137158. doi: 10.1371/journal.pone.0137158 (PMC4575104; doi:10.1371/journal.pone.0137158)
Supplement: S1 Text — (DOCX) [file pone.0137158.s001.docx]

S1 Text: Reporting Format

**MSF-OCA MEDICAL ERROR REPORT**

| **Reported By:** |  | **Date:** |  |
| --- | --- | --- | --- |
| **Country:** |  | **Project/health facility:** |  |

**DETAILS**

**---------------------------------------------------------------------------------**

- **DATE AND TIME:** (Describe when the error occurred as precisely as possible.)
- **RESPONSIBLE HEALTH CARE WORKER:**
- **DESCRIPTION OF THE ERROR:** (Describe as precisely as possible what went wrong)
- **Patient age and sex**
- **Diagnosis and treatment**
- ***How was the error discovered/intercepted?***
- **Patient outcome:** (harm caused by the error)
- **Generic and brand names of all medication involved:**
- **IMMEDIATE ACTIONS TAKEN:** (Detail any decisions and actions taken immediately following the error.)
- **WHO HAS BEEN INFORMED AND WHEN:** (Detail to whom the error has been reported to: patient / family / MSF-staff / MoH. Give time and date of notification.)
- **WHAT IS THE ANALYSIS OF ROOT CAUSE OF THE ERROR:**
- **FURTHER ACTIONS TO BE TAKEN:** (Detail the decisions and actions to be undertaken in response to the error. State any recommendations for improving patient safety and prevention of future similar errors.)

**Headquarters Feedback:**

- - All appropriate action taken
  - Requires further review at HQ level
  - Additional action required either for mission or organisation
