# Supplementary material for: Learning without Borders: A Review of the Implementation of Medical Error Reporting in Médecins Sans Frontières
Source: PLoS One. 2015 Sep 18;10(9):e0137158. doi: 10.1371/journal.pone.0137158 (PMC4575104; doi:10.1371/journal.pone.0137158)
Supplement: S2 Text — (DOCX) [file pone.0137158.s002.docx]

**S2 Text: List of resources to support field implementation**

**Incident Reporting**

- WHO Patient Safety Site: **<http://www.who.int/patientsafety/en/>**
- WHO Patient Safety Curriculum: **Topic 5. Learning from Errors to prevent harm.**
- Institute of Medicine: To err is human: building a safer health system. 1999. <http://www.nap.edu/html/to_err_is_human/reportbrief.pdf>
- WHO Draft Guidelines for Adverse Event Reporting and Learning Systems: http://www.who.int/patientsafety/implementation/reporting_and_learning/en/.

**Disclosure**

- Communicating with your patient about harm: Disclosure of Adverse Events. Canadian Medical Protective Association:

[**http://www.cmpa-acpm.ca/cmpapd04/docs/resource_files/ml_guides/disclosure/pdf/com_disclosure_toolkit-e.pdf**](http://www.cmpa-acpm.ca/cmpapd04/docs/resource_files/ml_guides/disclosure/pdf/com_disclosure_toolkit-e.pdf) **(English)**

[**http://www.cmpa-acpm.ca/cmpapd04/docs/resource_files/ml_guides/disclosure/pdf/com_disclosure_toolkit-f.pdf**](http://www.cmpa-acpm.ca/cmpapd04/docs/resource_files/ml_guides/disclosure/pdf/com_disclosure_toolkit-f.pdf) **(French)**

- Banja J. “Implementing a Process for Disclosure.” University Health System Consortium Conference on Clinical and Economic Imperative for Patient Safety. (October 2001)
- Wu A. et al. “To Tell the Truth: Ethical and Practical Issues in Disclosing Medical Mistakes to Patients.” Journal of General Internal Medicine. 12.12(December12, 1997):770-775

**Root Cause Analysis**

The London Protocol. Systems Analysis of clinical incidents: http://www1.imperial.ac.uk/cpssq/cpssq_publications/resources_tools/the_london_protocol/

**Patient Safety Tools**

1. World Health Organisation. Surgical Safety Checklist. Available at: <http://whqlibdoc.who.int/publications/2009/9789241598590_eng_Checklist.pdf?ua=1>.
2. WHO hand hygiene self assessment framework. http://www.who.int/gpsc/country_work/hhsa_framework_October_2010.pdf?ua=1
